# Insight on Exercise-Induced Heart Remodeling in Different Track and Field Disciplines

**DOI:** 10.3390/jcm13206027

**Published:** 2024-10-10

**Authors:** Giuseppe Di Gioia, Armando Ferrera, Francesca Vespasiano, Viviana Maestrini, Sara Monosilio, Erika Lemme, Andrea Serdoz, Federica Mango, Emanuele Casciani, Antonio Pelliccia, Maria Rosaria Squeo

**Affiliations:** 1 Institute of Sports Medicine and Science, National Italian Olympic Committee, Largo Piero Gabrielli, 1, 00197 Rome, Italy; dottgiuseppedigioia@gmail.com (G.D.G.); armando.ferrera95@gmail.com (A.F.); viviana.maestrini@uniroma1.it (V.M.); sara.monosilio@uniroma1.it (S.M.); erikalemme@msn.com (E.L.); andreaserdoz@gmail.com (A.S.); federicamango.md@gmail.com (F.M.); emanuelecasciani@gmail.com (E.C.); ant.pelliccia@gmail.com (A.P.); 2 Department of Movement, Human and Health Sciences, University of Rome “Foro Italico”, Piazza Lauro De Bosis, 15, 00135 Rome, Italy; 3 Clinical and Molecular Medicine Department, Sapienza University of Rome, 00198 Rome, Italy; 4 Department of Cardiovascular Sciences, Fondazione Policlinico Campus Bio-Medico di Roma, Via Alvaro del Portillo, 200, 00128 Rome, Italy; francesca.vespasiano@unicampus.it; 5 Department of Clinical, Internal, Anesthesiologic and Cardiovascular Sciences, Sapienza University of Rome, Piazzale Aldo Moro, 5, 00185 Rome, Italy; 6 Diagnostic Imaging Unit, Department of Biomedicine and Prevention, University of Rome “Tor Vergata”, 00133 Rome, Italy

**Keywords:** athletics, athlete’s heart, cardiac remodeling, Olympic, echocardiography, foot racing, sprints, exercise intensity, metabolic demands

## Abstract

**Background**: The foot racing disciplines include sprints, middle distances, and long distances, which vary in terms of intensities, duration of training, and metabolic demands. The aim of our study was to evaluate the differences in morpho-functional parameters describing cardiac remodeling in a large cohort of Olympic athletes practicing the different track subspecialties. **Methods**: We evaluated 140 track and field (52.1% males, mean age 26.3 ± 4.3 years) Olympic athletes divided into four groups according to the distance performed: Group A (46, 32.9%): 100 and 200 mt; Group B (34, 24.3%): 400 mt; Group C (25, 17.9%): 800, 1500, and 3000 mt; Group D (35, 24.9%): 5000, 10,000 mt, and marathon distance. The athletes underwent a pre-participation screening, which included transthoracic echocardiography and exercise stress testing. **Results**: In Group A and in Group B, most athletes presented normal cardiac geometry (41/46, 89.1% in Group A and 31/34, 91.2% in Group B, *p* < 0.0001). Instead, in Groups C and D, more than half presented eccentric cardiac remodeling (13\25, 52% in Group C and 23\35, 65.7% in Group D). No significant differences were found between subspecialties in LVEF (*p* = 0.587), diastolic function (*p* = 0.431), and training hours/week (*p* = 0.078). **Conclusions**: In conclusion, the presence and extent of cardiac remodeling vary according to the distance of the discipline practiced, with the largest dimensional increase in both left and right ventricles and atria in mid- and long-distance runners and the lowest in sprinters.

## 1. Introduction

Intense and prolonged physical exercise leads to a specific adaptation of the heart commonly known as “athlete’s heart” [1]. Differences in cardiac remodeling have been observed depending on the type of exercise training. Specifically, it has been reported that dynamic exercise leads to increased stroke volume, decreased vascular resistance, and enlargement of the heart chambers, refs. [2,3,4,5,6,7,8]. Conversely, static exercise results in increased transient blood pressure and is characterized by minimum thickening of the left ventricular (LV) wall without an accompanying increase in cavity dimensions [9,10]. This concept, known as the “Morganroth hypothesis”, describes distinct cardiac adaptations that occur in response to different types of training (endurance vs. resistance) [11]. While it has been a foundational concept in exercise physiology, the recent literature suggests that these adaptations are more gradual and nuanced than originally proposed [12].

Track and field is a sport that includes different contests based on running, jumping, and throwing skills with a wide range of hemodynamic loads and functional demands [13]. The foot racing disciplines include sprints (100, 200, and 400 m), middle distances (800, 1500, and 3000 m), and long distances (5000, 10,000 m, and marathon), which vary in terms of intensities, duration of training, and metabolic demands. Indeed, power disciplines (100, 200, and 400 m) primarily rely on the anaerobic energy system (both the anaerobic lactic and anaerobic lactic systems). In fact, these disciplines depend mainly on creatine phosphate and muscle glycogen as fuel for energy production with minimal oxygen use [14,15]. So, power athletes develop muscular strength, hypertrophy, and neuromuscular efficiency to generate maximum force in minimal time. In contrast, endurance disciplines such as middle distances and long distances predominantly rely on the aerobic energy system, which provides a sustained energy supply for prolonged activities depending largely on carbohydrates and fats as energy substrates. Endurance athletes develop a high level of aerobic capacity (VO2 max), which is the body’s ability to efficiently transport and utilize oxygen during prolonged activity, leading to improved cardiovascular and respiratory efficiency [16].

Both types of sports impose very different demands on the body, leading to specialized adaptations in terms of energy systems, muscle function, and recovery needs [14,17].

The aim of our study was to evaluate the differences in morpho-functional parameters describing cardiac remodeling in a large cohort of Olympic athletes practicing the different track subspecialties.

## 2. Materials and Methods

The Institute of Sport Medicine and Science in Rome functions as the medical division under the supervision of the Italian National Olympic Committee (CONI). Its primary objective is to carry out medical evaluations for athletes chosen to compete in prestigious events such as the Olympic Games, World Championships, and Mediterranean Games. The research methodology employed in this investigation underwent scrutiny and approval by the Review Board of the Institute of Medicine and Sports Science, date of approval 20 February 2023, approval code CNI200223f. All athletes participating in this study were fully informed about the nature and scope of the evaluation and gave their informed consent in accordance with Italian law and institutional protocols. The clinical data from this cohort are securely stored in an institutional database. The activities detailed herein were conducted in accordance with the Code of Ethics of the World Medical Association (Declaration of Helsinki).

We enrolled retrospectively 140 Olympic athletes practicing athletics (running) who participated at the London 2012, Rio 2016 and Tokyo 2020 Summer Olympic Games.

The athletes participated in an extensive, multidisciplinary pre-participation assessment that involved a thorough clinical examination, resting electrocardiography (ECG), transthoracic echocardiography (TTE), and a maximal exercise stress test.

Athletes who have arterial hypertension, diabetes (both type 1 and type 2), and/or cardiovascular structural or functional abnormalities were excluded.

Athletes were arbitrarily divided into 4 groups according to the main distance performed: Group A: 100 mt and 200 mt; Group B: 400 mt; Group C: 800 mt, 1500 mt, and 3000 mt; Group C: 5000 mt, 10,000 mt, and marathon distance.

Blood pressure was measured in the seated position prior to the exercise test, following the recommended guidelines [18].

Body height and weight were obtained in each subject, and body mass index (BMI) was calculated as weight (kg)\height (m^2^). Body surface area (BSA) was derived by the Mosteller formula [19]. Body composition and fat mass percentage were measured using bioelectric impedance analysis (BIA 101 Quantum, Akern, Italy) using constant sinusoidal current at an intensity of 50 kHz and 400 μA. Standard 12-lead ECG was performed in a supine position, and interpretations were made according to the international criteria for ECG interpretation in athletes [20]. All participants underwent maximal exercise testing on a bicycle ergometer (CubestressXR400; Cardioline S.p.A., Milan, Italy) as previously reported, with an incremental protocol until exhaustion.

### 2.1. Transthoracic Echocardiogram

The echocardiographic assessment was performed on athletes in a resting state, set in the left lateral decubitus orientation.

Ultrasound data acquisition was performed utilizing a GE Vivid E9 ultrasound system equipped with a 4Vc phased array probe (GE Healthcare Vingmed Ultrasound AS, Horten, Norway). A comprehensive 2D echocardiographic study was carried out, wherein cardiac images were captured in various cross-sectional planes employing established transducer positions. According to current recommendations [21], measurements of LV end-diastolic diameter (LVEDD), left ventricle end-systolic diameter (LVESD), interventricular septum (IVS) thickness, and PWT (posterior wall thickness) were taken in the parasternal short-axis section of the LV. The relative wall thickness (RWT) was calculated using the ratio of (IVS + PWT) to LVEDD [21]. LV mass (LVM) was derived using the Devereux formula [22], with measurements indexed to body surface area (BSA) [23]. An LVM indexed (LVMi) > 115 g/m^2^ for males and >95 g/m^2^ for females was indicative of LV hypertrophy [23]. Different types of LV remodeling [21] were defined based on the measurements obtained, including normal geometry (NG), defined as LVM ≤ 115 g/m^2^ in males or ≤95 g/m^2^ in females and RWT ≤ 0.42; concentric remodeling (CR), as LVM ≤ 115 g/m^2^ in males or ≤95 g/m^2^ in females and RWT > 0.42; concentric hypertrophy (CH), as LVM > 115 g/m^2^ in males or >95 g/m^2^ in females and RWT > 0.42; and eccentric remodeling (ER), as LVM > 115 g/m^2^ in males or >95 g/m^2^ in females and RWT ≤ 0.42.

LV systolic function was assessed based on LV ejection fraction (EF), derived from LV end-diastolic volume (LVEDV) and end-systolic volume (LVESV) computed through LV biplane planimetry employing the modified Simpson’s rule in both the apical 2- and 4-chamber views [23]. Diastolic function was evaluated using both pulsed-wave Doppler (PW) and tissue Doppler imaging (TDI) as recommended [24], based on the measurement of maximum blood flow velocities (Vmax) of E- and A-wave, E/A-ratio, as well as myocardial Vmax of e′ and a′ at the basal septal and lateral tricuspid annulus, along with the septal E/e′-ratio [24]. Left atrial (LA) volume was determined using the biplane method. The right ventricular (RV) chamber was evaluated in accordance with established guidelines [6]. The right atrial (RA) area and right ventricular (RV) function parameters were assessed using the RV-focused apical four-chamber view. The endocardial contour of the end-diastolic and systolic areas was traced, and from these measurements, the fractional area change (FAC) was calculated and expressed as a percentage [6]. Tricuspid annular plane systolic excursion (TAPSE) was measured as an indicator of RV longitudinal systolic function [6]. Peak tricuspid regurgitant velocity and the systolic trans-tricuspid gradient were assessed using continuous wave Doppler on the tricuspid regurgitation jet. Pulmonary artery systolic pressure (PASP) was calculated by adding the systolic trans-tricuspid gradient to the value of right atrial pressure (RAP). The latter was estimated using the inferior vena cava (IVC) dimension, inspiratory collapsibility, and RV function [6]. In all athletes, three consecutive cardiac cycles were assessed in accordance with the current literature [25].

All echocardiographic measurements were conducted by skilled sports cardiologists (GDG and AP). Between 2012 and 2016, only one expert sports cardiologist (AP) performed the echocardiograms. From 2016 to 2022, a second physician (GDG) joined the team and began conducting echocardiograms. Nevertheless, all reports were re-evaluated by AP prior to validation.

Intra-observer and inter-observer variability was assessed. Two investigators (GDG and AP), blinded, measured the same exam. Both investigators repeated the analysis two days later, without knowledge of the previous measurements. The interclass correlation coefficients (ICCs) for LVEF were 0.91 for intra-observer and 0.92 for inter-observer agreement; for LVEDD, the ICCs were 0.93 for intra-observer and 0.92 for inter-observer agreement; for IVS thickness, the ICCs were 0.94 for intra-observer and 0.93 for inter-observer agreement; for LVEDV, the ICCs were 0.90 for intra-observer and 0.89 for inter-observer agreement. Discrepancies among observers were resolved through consensus.

### 2.2. Statistical Analysis

Categorical variables were reported as frequencies and percentages and analyzed using either Fisher’s exact test or the Chi-square test, depending on the situation. For continuous variables, normality was assessed, and results were presented as means with standard deviations (SDs), with comparisons made using the Student’s *t*-test for independent samples if the data followed a normal distribution. Pearson’s correlation coefficient was utilized for correlation analyses. A significance level of *p* < 0.05 was applied to all tests. Intraclass correlation coefficients (ICCs) were calculated to evaluate both inter-observer and intra-observer agreement regarding the primary left ventricle measurements. The statistical analysis was conducted using STATA Statistics for Windows (SE, version 17).

## 3. Results

Of the enrolled 140 athletes, 73 were males (52.1%), with a mean age of 26.3 ± 4.3 years and a mean BMI of 20.4 ± 2.6 kg/m^2^. Based on the type of discipline, we classified 46 athletes (32.9%) into a 100 mt and 200 mt group (Group A), 34 athletes (24.3%) into a 400 mt group (Group B), 25 athletes (17.9%) into an 800 mt, 1500 mt, and 3000 mt group (Group C), and 35 athletes (24.9%) into a 5000 mt, 10,000 mt, and marathon distance group (Group D). In Table 1, clinical and anthropometric parameters in different disciplines are listed. No significant gender differences were present, with a similar prevalence of male athletes (*p* = 0.511). Athletes performing long distance running (5000, 10,000, and marathons) were older (29 ± 5.1) compared to other groups (24.8 ± 3.6 years old in Group A, 25.6 ± 3.7 years old in Group B, and 26 ± 3.4 years old in Group C; *p* < 0.0001). Other differences were found in anthropometric characteristics, with a progressive reduction in body weight (*p* < 0.0001), BMI (*p* < 0.0001), and BSA (*p* < 0.0001) from Group A (sprinters) to Group D (long-distance runners).

**Figure 1:**
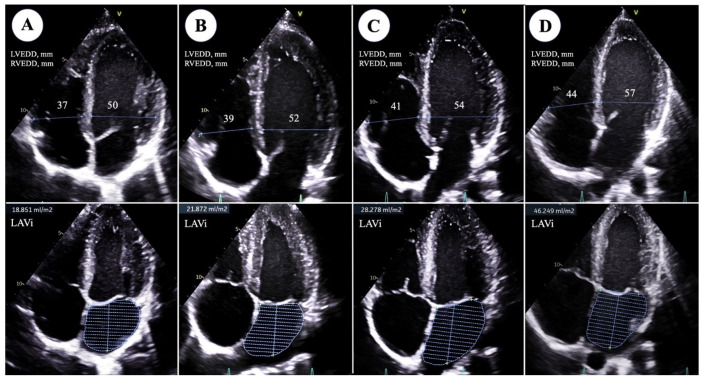
Echocardiographic comparison of main morphological parameters (left ventricle end-diastolic diameter, LVEDD; right ventricle end-diastolic diameter, RVEDD; LAVi: left atrial volume indexed) between athletes practicing different track and field subspecialties: (**A**) 100–200 mt; (**B**) 400 mt; (**C**) 1500 mt; (**D**) marathon.

Peculiar differences in echocardiographic measurements are highlighted (Table 2). A cardiac remodeling was observed, with multiparametric significant differences evident from Group A to Group D, including chamber size enlargement (both linear dimensions and volumes) of both ventricles and atria: i.e., LVEDD indexed (LVEDDi) (ranging from 27.3 ± 2 mm/m^2^ in Group A to 32.5 ± 2.3 mm/m^2^ in Group D), *p* < 0.0001; LVESD indexed (LVSEDi) (from 17.2 ± 1.9 mm/m^2^ in Group A to 19.5 ± 2.1 mm/m^2^ in Group D), *p* < 0.0001; LVEDV indexed (LVEDVi) (from 59 ± 13.1 mL/m^2^ in Group A to 81.5 ± 16.3 mL/m^2^ in Group D); IVS thickness (ranging from 9.3 ± 1 mm in Group A to 9.6 ± 1.1 mm in Group D), *p* = 0.008; PWT (from 8.7 ± 1 mm in Group A to 9.3 ± 1 mm in Group D), *p* = 0.003; LVM indexed (from 86.3 ± 16.2 g/m^2^ in Group A to 113.8 ± 19.2 g/m^2^ in Group D), *p* < 0.0001; LAV indexed (LAVi) (ranging from 18.1 ± 6 mL/m^3^ in Group A to 25.1 ± 9.3 mL/m^3^ in Group D), *p* < 0.0001; and RVOT indexes (RVOTi) LAX (*p* < 0.0001), RVOT indexed (RVOTi) SAX (*p* < 0.0001), and right area (RA) (*p* = 0.003), Figure 1. No significant differences, instead, were observed among groups regarding indexes of systolic function (*p* = 0.587) and diastolic function (E/E’, *p* = 0.431).

We then calculated the prevalence of different LV geometry in the overall athletes’ population (Table 3). In the group performing short distances (100 mt, 200 mt, and 400 mt), the majority of athletes presented a normal geometry (41/46, 89.1% in Group A and 31/34, 91.2% in Group B), *p* < 0.0001. Instead, in the group practicing mid-to-long distance (from 800 mt to marathon, Groups C and D), more than half presented an eccentric remodeling (13\25, 52% in Group C and 23\35, 65.7% in Group D). Of notice, no cases of concentric remodeling were highlighted, and only one athlete in Group D had concentric hypertrophy.

**Figure 2:**
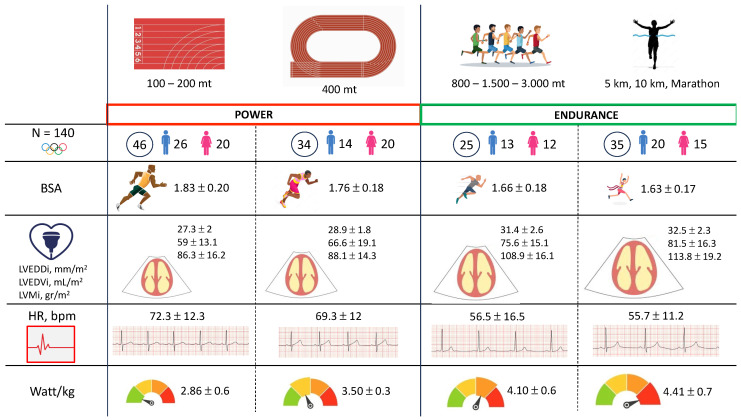
Summary of main anthropometric, morphological and functional heart remodeling differences between athletes practicing different track and field subspecialties. Abbreviations: BSA: body surface area; HR: heart rate; LVEDDi: left ventricle end-diastolic diameter indexed; LVEDVi: left ventricle end-diastolic diameter indexed; LVMi: left ventricle mass indexed.

Finally, differences in the exercise stress test are reported in Table 4. Also in functional parameters, a tendency towards increasing adaptive mechanisms, according to the main distance, was found. In fact, HR was progressively lower from Group A to Group D (ranging from 72.3 ± 12.3 bpm to 55.7 ± 11.2 bpm, *p* < 0.0001). On the other hand, Groups C and D showed the highest functional capacity (Watt/kg 4.1 ± 0.6 in Group C and 4.41 ± 0.7 in Group D) compared to the remaining disciplines (Group A 2.86 ± 0.6 Watt/kg and Group B 3.50 ± 0.3, *p* < 0.0001). No differences were found in blood pressure profile both at rest (SBP, *p* = 0.619; DBP, *p* = 0.546) and at the exercise peak (SBP, *p* = 0.722; DBP, *p* = 0.365). Moreover, no significant arrhythmic burden was found between the four different groups (VEB, *p* = 0.560; SVEB, *p* = 0.227) Figure 2.

## 4. Discussion

Our study aimed to evaluate differences in morpho-functional parameters of cardiac remodeling in Olympic athletes practicing different subspecialties of track and field athletics. The drive for this analysis was prompted by the recognition that different disciplines in athletes should have a variable impact on cardiac remodeling, based on the differences in terms of energy demands and hemodynamic involvement, which are so different from 100 m to marathon. In fact, in Group A (sprinters), athletes perform short, intense efforts, predominantly anaerobic, while in Group D (long-distance runners), athletes sustain a prolonged effort, requiring a constant supply of oxygen to facilitate aerobic muscle work.

As the distance increased in sporting disciplines, we observed a gradually increasing remodeling of the heart, outlined by significant changes, mostly exemplified by a progressive increase in the size of all chambers (both ventricles and atrias). Dynamic aerobic exercise, in fact, leads to an increase in stroke volume, with a reduction in systemic vascular resistance, determining a significant increase in cardiac output [8,26]. The heart adapts by increasing the internal diameter and wall thickness of the left ventricle, developing an eccentric remodeling [27]. Accordingly, our study showed that long distances were associated with significant enlargement of the left and right ventricles.

Moreover, we also evaluated differences in performance parameters, underscoring a tendency towards increasing adaptive functional mechanisms, according to the main distance practiced. Actually, heart rate was progressively lower from Group A to Group D, with Groups C and D showing the highest functional capacity (Watt/Kg at exercise testing) compared to disciplines in Groups B and A. The differences in cardiac remodeling can be physiologically explained through several mechanisms related to the specific training stimulus, considering that the characteristics of intensity and duration of hemodynamic load vary between different sports subspecialties, and in certain disciplines, athletes combine parts of static and dynamic hemodynamic loads [28].

Since the first echocardiographic definition in the 1970s, athletes’ hearts have been characterized dichotomously: endurance athletes with increased “volume load” vs. strength athletes with “pressure load” [29], as per the “Morganroth hypothesis” [29]. However, while this hypothesis is physiologically attractive, it is likely an oversimplified representation of cardiac remodeling in response to exercise training. Our study challenges this dichotomous theory showing a gradual adaptation of the heart in relation to the distances practiced by athletes. Moreover, we did not find, different from the “Morganroth hypothesis”, that high-intensity isometric activity (such as in 100 m) leads to concentric hypertrophy as a response to the pressure overload on the ventricle [29]. Additionally, in our study, none of the athletes exhibited diastolic dysfunction, contrary to Morganroth et al.’s findings that reported increased ventricular stiffness [29]. Similarly to our study, Coates et al. compared the sport-specific cardiac structure of elite aquatic sports, showing similar sport-specific differentiation in cardiac remodeling [30]. Our present investigation could contribute to better understanding the specific cardiac remodeling associated with different athletic disciplines, which is crucial for accurate interpretation of cardiac imaging in athletes. In fact, misclassifying physiological adaptations as pathological can lead to unnecessary restrictions and anxiety for athletes. Our study has some strengths, such as a large sample size, the use of robust diagnostic tools such as echocardiography and exercise stress testing, and the consideration of intra- and inter-observer agreement in the clinical analysis. Furthermore, Olympic athletes provide insights into cardiac adaptations at the highest levels of performance. These individuals often exhibit unique physiological characteristics that are relevant to understanding elite athletic training effects. Moreover, analyzing athletes from various sports disciplines allows for comparisons of how different training modalities (e.g., endurance vs. power sports) influence cardiac structure and function. This comparative analysis can help elucidate specific adaptations relevant to each sport. Furthermore, to our knowledge, this is the first study in the literature to describe cardiac remodeling in the different disciplines of track and field athletics. By filling gaps in current research regarding cardiac health in elite athletes, this study can inform future investigations and influence training practices. Contributions to the scientific literature are crucial for advancing knowledge in sports medicine and exercise physiology.

## 5. Limitations

Our study has several limitations. Firstly, it is designed as a retrospective study, meaning that data collection and analysis were based on previously obtained information. Secondly, although athletes were evaluated during a training period, the seasonal variability was not specifically considered for each individual athlete. It is reasonable to assume that the athletes adjusted their training load throughout the season, which differs from one discipline to another, thereby representing a limiting factor. Finally, although this research focused on Italian Olympic athletes who undergo rigorous and ongoing anti-doping testing, previous studies have linked track and field athletes with a higher incidence of doping cases [31,32]. While this study does not specifically address the use of performance-enhancing substances, it is important to recognize that such practices could complicate the interpretation of cardiac remodeling patterns. Nevertheless, the potential influence of performance-enhancing substance use on an athlete’s heart is an important factor that should be further explored.

## 6. Conclusions

Our study shows that among athletes engaged in track and field, the presence and extent of cardiac remodeling vary according to the distance of the discipline practiced, with the largest dimensional increase in both left and right ventricles and atria in mid- and long-distance runners and the lowest in sprinters. No differences, instead, were observed regarding indexes of systolic and diastolic relations and filling. A large majority of mid- and long-distance runners, but no sprinters, showed eccentric LV remodeling. Functional performance (as efficiency on exercise testing) and hemodynamic response to effort were consistently superior in long-distance runners compared to sprinters.

Our data suggest that cardiac remodeling in response to athletic training is a complex mechanism that varies in response to the type and intensity of exercise performed.

## Figures and Tables

**Table 1:** Clinical, anthropometric, and demographic characteristics in different disciplines.

N = 140	100–200 mt	400 mt	800–1500–3000 mt	5000–10,000 mt–Marathon	
N, (%)	46 (32.9)	34 (24.3)	25 (17.9)	35 (25)	
Male, n (%)	26 (56.5)	14 (41.8)	13 (52)	20 (57.1)	0.511
Age, years	24.8 ± 3.6	25.6 ± 3.7	26 ± 3.4	29 ± 5.1	0.0001
Afro-Caribbean, n (%)	12 (26.1)	6 (17.6)	8 (32)	6 (17.1)	0.459
Familiarity for CVD, n (%)	9 (19.6)	6 (17.6)	2 (8)	9 (25.7)	0.388
Weight, kg	69.8 ± 11.5	64.3 ± 10.8	58.9 ± 9.3	55.9 ± 9.3	<0.0001
BSA	1.83 ± 0.20	1.76 ± 0.18	1.66 ± 0.18	1.63 ± 0.17	<0.0001
BMI, kg/m^2^	21.9 ± 3.1	20.7 ± 2.2	19.5 ± 1.5	19 ± 1.8	<0.0001
Fat mass, %	11.4 ± 4.9	12.1 ± 4.4	12 ± 4.8	10.8 ± 4.9	0.677
Training hours per week	19.3 ± 6.2	18 ± 5.2	19.1 ± 5.7	22.6 ± 7.5	0.078

BMI: body mass index; BSA: body surface area; CVD: cardiovascular disease.

**Table 2:** Echocardiographic differences in athletes practicing different athletic disciplines.

N = 140	100–200 mt	400 mt	800–1500–3000 mt	5000–10,000 mt–Marathon	
N, (%)	46 (32.9)	34 (24.3)	25 (17.9)	35 (25)	
LVEDDi, mm/m^2^	27.3 ± 2	28.9 ± 1.8	31.4 ± 2.6	32.5 ± 2.3	<0.0001
LVESDi, mm/m^2^	17.2 ± 1.9	17.8 ± 1.5	19.7 ± 1.9	19.5 ± 2.1	<0.0001
LVEDVi, mL/m^2^	59 ± 13.1	66.6 ± 19.1	75.6 ± 15.1	81.5 ± 16.3	<0.0001
LVESVi, mL/m^2^	21.4 ± 6.4	23.8 ± 8.7	26.7 ± 6.1	28.1 ± 8	0.0008
IVS, mm	9.3 ± 1	8.9 ± 1.1	9.6 ± 0.8	9.6 ± 1.1	0.008
PWT, mm	8.7 ± 1	8.6 ± 1	9.3 ± 0.9	9.3 ± 1	0.003
LVMi, g/m^2^	86.3 ± 16.2	88.1 ± 14.3	108.9 ± 16.1	113.8 ± 19.2	<0.0001
EF, %	64.3 ± 5.6	64.6 ± 6.1	63.9 ± 4.9	65.8 ± 5.4	0.587
LAD, mm	32.9 ± 3.5	33.5 ± 3.6	35.6 ± 3.4	36.3 ± 3.3	<0.0001
LAVi, mL/m^3^	18.1 ± 6	17 ± 3.1	22.4 ± 5.4	25.1 ± 9.3	<0.0001
AR, mm	29.1 ± 3.5	29.1 ± 3.5	29.2 ± 3.3	29.6 ± 2.6	0.930
AA, mm	25.3 ± 2.9	25.7 ± 2	25.9 ± 1.8	27.4 ± 3.1	0.042
E wave, cm/sec	82.7 ± 16.1	86.4 ± 15.2	82.2 ± 12.3	84.1 ± 13.4	0.663
A wave, cm/sec	48.6 ± 10.8	45.3 ± 11.7	42.4 ± 8.5	43.6 ± 10.5	0.082
E/A	1.77 ± 0.5	2.03 ± 0.6	2 ± 0.4	2.03 ± 0.5	0.083
E’, m/sec	12.5 ± 2.4	12.4 ± 2.5	11.9 ± 1.7	11.7 ± 2.1	0.408
A’, m/sec	6.3 ± 1.3	6.1 ± 1	5.9 ± 1.2	6.4 ± 1.4	0.497
S’, m/sec	7.8 ± 1.3	7.8 ± 1.3	7.6 ± 1.4	8.1 ± 1.2	0.568
E/E’	6.79 ± 1.5	7.2 ± 2	7 ± 1.1	7.3 ± 1.6	0.431
PASP, mmHg	22.2 ± 3.2	23.1 ± 3.4	21.1 ± 4.7	24 ± 4.9	0.049
TAPSE, mm	25.3 ± 2.8	26.5 ± 3	26.3 ± 4	26.4 ± 4.5	0.430
RVEDA, mm^2^	21.1 ± 5	22.3 ± 5.3	24.2 ± 6.4	23 ± 4.4	0.204
RVESA, mm^2^	11.8 ± 3	12.7 ± 3.1	12.8 ± 3.6	11.8 ± 2.3	0.502
FAC, %	43.8 ± 6.1	43.1 ± 8	47.1 ± 5.4	48.7 ± 7.3	0.008
RVOTi LAX, mm^2^	15.3 ± 2.1	16.2 ± 2.3	18.2 ± 2.8	18.4 ± 2.5	<0.0001
RVOTi SAX, mm^2^	14.8 ± 1.9	15.3 ± 2.1	17.6 ± 2.5	17.6 ± 3.9	<0.0001
RVEDDi, mm^2^	18.7 ± 1.3	22.3 ± 1.2	23.6 ± 1.1	23.8 ± 0.4	0.049
RAA, mm^2^	6.9 ± 1.4	8.1 ± 1.6	12.7 ± 3	11.6 ± 2.1	0.003

AA: ascending aorta; AR: aortic root; EDA: end-diastolic area; EDDi: end-diastolic diameter indexed; EDVi: end-diastolic volume indexed; EF: ejection fraction; ESA: end-systolic area; ESDi: end-systolic diameter indexed; ESVi: end-systolic volume indexed; FAC: fractional area change; IVS: interventricular septum; LA: left atrium; LAVi: left atrium volume indexed; LAX: long axis; LV: left ventricle; LVMi: left ventricular mass indexed; PASP: pulmonary artery systolic pressure; PWT: posterior wall thickness; RV: right ventricle; RVOT: right ventricle outflow tract; TAPSE: tricuspid annular plane systolic excursion.

**Table 3:** Different heart remodeling according to athletic subspecialties.

N = 140	100–200 mt	400 mt	800–1500–3000 mt	5000–10,000 mt–Marathon	
N, (%)	46 (32.9)	34 (24.3)	25 (17.9)	35 (25)	
NG, n (%)	41 (89.1)	31 (91.2)	12 (48)	11 (31.4)	<0.0001
EH, n (%)	5 (10.9)	3 (8.8)	13 (52)	23 (65.7)	<0.0001
CR, n (%)	0 (0)	0 (0)	0 (0)	0 (0)	NS
CH, n (%)	0 (0)	0 (0)	0 (0)	1 (2.9)	NS

CH: concentric hypertrophy; CR: concentric remodeling; EH: eccentric hypertrophy; NG: normal geometry.

**Table 4:** Main differences in exercise stress tests in athletes practicing different athletic disciplines.

N = 140	100–200 mt	400 mt	800–1500–3000 mt	5000–10,000 mt–Marathon	
N, (%)	46 (32.9)	34 (24.3)	25 (17.9)	35 (25)	
Rest HR, bpm	72.3 ± 12.3	69.3 ± 12	56.5 ± 16.5	55.7 ± 11.2	<0.0001
Peak HR, bpm	166.6 ± 9.5	167 ± 8.8	158.5 ± 8.5	157.5 ± 13.9	<0.0001
Rest SBP, mmHg	108.7 ± 10	105.2 ± 12.1	105 ± 12.2	106.2 ± 15.1	0.619
Rest DPB, mmHg	67.1 ± 7.4	67.8 ± 7.6	69.5 ± 7.1	66.5 ± 8	0.546
Peak SBP, mmHg	173.3 ± 17.9	171.7 ± 19.8	176.2 ± 19.7	170.3 ± 23.4	0.722
Peak DPB, mmHg	71.8 ± 6.2	72.7 ± 8.3	71.6 ± 7.4	74.6 ± 8.3	0.365
Watt max	205.1 ± 45.4	224.6 ± 43.9	239.6 ± 39.1	245.6 ± 43.4	0.0004
Watt max/kg	2.86 ± 0.6	3.50 ± 0.3	4.1 ± 0.6	4.41 ± 0.7	<0.0001
SVEB, n (%)	0 (0)	3 (8.8)	1 (4)	1 (2.8)	0.227
VEB, n (%)	5 (10.9)	5 (14.7)	4 (16)	5 (14.3)	0.560

DBP: diastolic blood pressure; HR: heart rate; SBP: systolic blood pressure; SVEB: supra-ventricular ectopic beats; VEB: ventricular ectopic beats.

## Data Availability

Anonymized participant data can be accessed upon reasonable request by contacting the corresponding author.
